# Antimicrobial Susceptibility Profiles of *Acinetobacter baumannii* Strains, Isolated from Clinical Cases of Companion Animals in Greece

**DOI:** 10.3390/vetsci10110635

**Published:** 2023-10-29

**Authors:** Marios Lysitsas, Eleutherios Triantafillou, Irene Chatzipanagiotidou, Konstantina Antoniou, George Valiakos

**Affiliations:** 1 Faculty of Veterinary Science, University of Thessaly, 43100 Karditsa, Greece; lysitsas91@gmail.com; 2 Vet Analyseis, Private Diagnostic Laboratory, 41335 Larissa, Greece; eltriantafil@gmail.com (E.T.); antonioukwn@gmail.com (K.A.); 3 Department of Biochemistry and Biotechnology, University of Thessaly, 41500 Larissa, Greece; irenechatzi1@hotmail.com

**Keywords:** *Acinetobacter baumannii-calcoaceticus* complex, companion animals, polymicrobial infections, carbapenem resistant, XDR

## Abstract

**Simple Summary:**

*Acinetobacter baumannii* Complex is a worldwide distributed group of species responsible for several challenges in treating nosocomial infections in humans. The emergence of highly resistant strains, even to last-resort antibiotics, constitutes a severe threat, especially for hospitalized patients. In veterinary medicine, its role has not been comprehensively investigated yet. However, many recent studies indicate its ability to cause infections in multiple animal species, primarily pets. In this study, we obtained a significant number of *A. baumannii* isolates from canine and feline clinical samples during 2.5 years, in Greece. Data regarding the isolates’ sample origin, type of infection and resistance profile were collected and compared. High resistance rates against several antibiotics were detected, including agents of paramount clinical importance, such as carbapenems. This study indicates the emergence of *Acinetobacter baumannii* Complex bacteria as pathogens for companion animals, the prevalence of strains with acquired resistance to many of antibiotics, and the danger of circulation of these strains between animals, humans, and veterinary equipment and facilities.

**Abstract:**

*Acinetobacter baumannii–calcoaceticus* (*Abc*) Complex bacteria are troublesome nosocomial pathogens in human medicine, especially during the last 30 years. Recent research in veterinary medicine also supports its emergence as an animal pathogen. However, relevant data are limited. In this study, we obtained 41 *A. baumannii* isolates from clinical samples of canine and feline origin collected in veterinary clinics in Greece between 2020 and 2023. Biochemical identification, antimicrobial susceptibility testing, molecular identification and statistical analysis were performed. Most of the samples were of soft tissue and urine origin, while polymicrobial infections were recorded in 29 cases. Minocycline was the most effective in vitro antibiotic, whereas high resistance rates were detected for almost all the agents tested. Notably, 20 isolates were carbapenem resistant and 19 extensively drug resistant (XDR). This is the first report of canine and feline infections caused by *Abc* in Greece. The results create concerns regarding the capability of the respective bacteria to cause difficult-to-treat infections in pets and persist in veterinary facilities through hospitalized animals, contaminated equipment, and surfaces. Moreover, the prevalence of highly resistant strains in companion animals constitutes a public health issue since they could act as a reservoir, contributing to the spread of epidemic clones in a community.

## 1. Introduction

Bacteria of the genus *Acinetobacter* are members of the Moraxellaceae family, which were comprehensively described and recognized approximately 60 years ago [1,2]. They are Gram-negative, non-fastidious, strictly aerobic, catalase-positive, oxidase-negative bacteria [3], regularly associated with nosocomial infections in humans [4].

The majority of clinical infections caused by *Acinetobacter* spp. are attributed to a specific group of species, including *A. baumannii*, *A. nosocomialis*, *A. pittii*, *A. calcoaceticus*, *A. seifertii*, and *A. dijkshoorniae*. These species are usually referred to as *Acinetobacter baumannii-calcoaceticus* (*Abc*) complex, since they are closely related and their discrimination through phenotypic and biochemical characteristics is insufficient [3,4]. *Acinetobacter baumannii* is of major importance in human medicine over the last three decades [4]. It is unsurprisingly included in the ESKAPE bacteria (*Enterococcus faecium*, *Staphylococcus aureus*, *Klebsiella pneumoniae*, *Acinetobacter baumannii*, *Pseudomonas aeruginosa*, *Enterobacter* spp.), listed as one of the most challenging pathogens for health care professionals [5].

Bacteria of the *Abc* Complex are intrinsically resistant to a variety of agents, such as aminopenicillins (even combined with the β-lactamase inhibitor clavulanic acid), first- and second-generation cephalosporins, including cephamycins (cefoxitin and cefotetan), chloramphenicol and fosfomycin [6,7,8]. Furthermore, the acquisition of resistance against other classes of antibiotics is frequent, dramatically reducing the available treatment options in the armamentarium of health scientists [4]. For example, the worldwide emergence of carbapenem-resistant strains in the 21st century significantly limited the therapeutic strategies. Further, these strains were regularly resistant to several antibiotics, possibly due to the acquisition of mobile genetic elements containing numerous antibiotic resistance genes (ARGs) [3,5].

In veterinary medicine, data regarding the *Abc* bacteria’s distribution, virulence, and resistance profiles are limited [9]. However, several studies, especially during the last two decades, have provided evidence that they could constitute considerable animal pathogens [9,10,11,12,13].

The objective of this study was to demonstrate the emergence of *Abc* infections in companion animals and the distribution of highly resistant isolates in canine and feline populations in Greece. Moreover, it indicates the danger of persistent circulating relevant strains in veterinary facilities and between animals and their owners.

## 2. Materials and Methods

### 2.1. Isolation and Selection of the Bacterial Strains

The bacteria included in this study were obtained from canine and feline clinical samples received during October 2020–May 2023. These samples had been collected in veterinary clinics throughout Greece during routine veterinary practices. Isolation, phenotypic and biochemical identification as well as routine susceptibility testing were initially performed. The identification of the isolates belonging to the *Abc* complex included criteria provided by literature [14], such as Gram-negative, catalase-positive, oxidase-negative coccobacilli, non-hemolytic, opaque circular colonies on sheep blood agar (Figure S1), growth on MacConkey Agar (pink-grey opaque colonies (Figure S2), inhibition of growth in anaerobic conditions, Tryptic Sugar Iron (TSI) Agar profile: Alkaline slant/Alkaline butt/Gas (−)/H2S (−). Additional biochemical tests were performed by a commercial identification kit (Microgen GNA-ID System, Microgen Bioproducts Ltd.—Figure S3), evaluating the following assays: negative test for mannitol fermentation, indole production, nitrate reduction, and urease test, positive for glucose and citrate utilization. Isolates identified as members of the *Acinetobacter baumannii-calcoaceticus* complex were subsequently inoculated into a general-purpose culture medium (Tryptic Soy Agar) in order to achieve a pure culture, collected and maintained in Brain–Heart Infusion Broth supplemented with 20% glycerol at −80 °C.

### 2.2. Antimicrobial Susceptibility Testing

The identification of the resistance profiles of the isolates was initially performed through the disk diffusion method (Figure S4) and consequently confirmed, by the minimum inhibitory concentration (MIC) method (VITEK^®^2, bioMérieux, Lyon, France). Antibiotics were selected from the respective table of agents that should be considered for testing against *Acinetobacter* spp., of the relevant CLSI document [15], focusing on agents that could be utilized in veterinary medicine. Therefore, during antimicrobial susceptibility testing (AST), piperacillin + tazobactam, imipenem, amikacin, gentamicin, tobramycin, ciprofloxacin, sulfamethoxazole + trimethoprim and minocycline were evaluated with both techniques. Doxycycline ampicillin + sulbactam, ceftazidime, cefepime and enrofloxacin were tested only by the disc diffusion method, while meropenem, colistin, and levofloxacin were examined only by MIC.

For the disc diffusion method, a colony of each isolate was inoculated into saline, and the suspension was subsequently compared to a McFarland standard tube, to achieve the desirable turbidity (0.5 McFarland). The suspension was then vortexed, and a quantity was inoculated on the surface of Mueller–Hinton agar plates using a sterile swab. Susceptibility discs were added, and the plates were incubated at 35 °C for 16–18 h.

During the MIC evaluation, identification and AST were assessed by VITEK^®^2 GN ID and VITEK^®^2 AST-N376 cards, respectively (bioMérieux, Lyon, France).

The contents of the disks, the zone diameter and the MIC breakpoints, as specified by the CLSI document, are presented in Table 1.

### 2.3. PCR for Detection of the bla_OXA-51-like_ Gene

All isolates were subjected to PCR, to detect the *bla*_OXA-51-like_ gene, which is intrinsic in *Acinetobacter baumannii* [16].

Whole genomic DNA extraction was performed, from all the selected isolates, using a commercial spin-column kit (IndiSpin Pathogen Kit, INDICAL BIOSCIENCE GmbH). The procedures were carried out according to the manufacturer’s instructions. To perform PCR, the following primers were used: OXA-51-likeF 5′-TAA TGC TTT GAT CGG CCT TG-3′ and OXA-51-likeR 5′-TGG ATT GCA CTT CAT CTT GG-3′, as previously described [16]. Briefly, for the reaction, a 25 μL mix was created for each strain, by adding 12.5 μL of Xpert Fast Mastermix (2X) with dye (GRiSP Research Solutions, Porto, Portugal), 2 μL (10 pmols) of each primer, 0,5 μL of bacterial DNA and 8μL of PCR-grade water. the conditions were the following: 95 °C for 1 min, and then 40 cycles at 95 °C for 15 s (denaturation), at 60 °C for 15 s (annealing), and at 72 °C for 3 sec (elongation), followed by a final extension at 72 °C for 3 min. DNA products were identified after electrophoresis in 0.5 Tris-borate-EDTA using 1.5% agarose gel stained with ethidium bromide solution.

## 3. Results

### 3.1. Origin of the Isolates

A group of 41 isolates was obtained during the period mentioned above. Samples were received from 14 different veterinary clinics in four cities throughout the country (Athens, Thessaloniki, Serres, Larissa). All of them were identified as members of the *Abc* complex by the conventional biochemical tests. The bionumbers obtained by the VITEK^®^2 compact were 02(V_a_)1010(V_b_)03500(V_c_V_d_V_e_) (V_a_ = 0 or 4, V_b_ = 1 or 3, V_c_ = 2 or 3, V_d_ = 1 or 5, V_e_ = 0 or 2), providing an excellent identification of *Acinetobacter baumannii* with a 99% probability for all 41 strains. Data regarding the origin of the samples, animal species, and site of infection are presented in Table 2.

Canine and feline strains were mainly associated with soft tissue (mostly wounds and skin abscesses, 61.5%) and urine samples (53.3%), respectively, showing different distribution (chi^2^ = 12.48, df = 5, *p* = 0.028). A few bacteria originated from different sites of infection, such as the ear canal, nasal cavity, pleural effusions, and blood.

The characteristics of the infected animals and the bacterial species, which were co-isolated in the same samples, are presented in Table 3.

Approximately two-thirds (65.9%) of the infected animals (27/41) were males. However, the sample size was relatively small for a safe assumption. The average age of the infected animals was approximately 6.5 (SD = 3.8) years old. Regarding dogs, the mean age was 7.3 (SD = 3.8) years old, while the average age in cats was relatively lower, at approximately 5,2 (SD = 3.6) years old.

Moreover, apart from *Abc* complex strains, more bacterial species were co-isolated in a significant percentage of the samples. Polymicrobial infections were identified in 29 of 41 (70.7%) samples; 26 of 29 (89.7%) non-urine, 18 of 20 (90.0%) soft tissue and all four (100.0%) ear samples. More than one bacterial species were isolated from 20/26 (76.9%) canine and 9/15 (60.0%) feline samples.

Particularly, 17 members of the Enterobacteriaceae family were obtained from 16 of these samples (thirteen canine and three feline). These isolates include *E. coli* (*n* = 5), *Klebsiella pneumoniae* (*n* = 3), *Proteus mirabilis* (*n* = 3), *Enterobacter cloacae* (*n* = 3), *Klebsiella oxytoca* (*n* = 1), *Klebsiella aerogenes* (*n* = 1) and *Pluralibacter gergoviae* (*n* = 1). Twelve of these strains were MDR. Methicillin-resistant Staphylococci (*S*. *pseudintermedius*, *S. aureus*, *S. epidermidis*) were also obtained from nine samples (seven canine and two feline), all except one of soft tissue origin.

In contrast, *Acinetobacter baumannii* was the only bacterial species obtained in 9/12 urine samples (75.0%).

### 3.2. Antimicrobial Susceptibility Testing

Detailed results of the susceptibility testing of the isolates by disc diffusion, are available in Table S1 (Supplementary File). Data regarding the prevalence of resistance for each antibiotic, by the disc diffusion method, are presented in Table 4. All strains (41/41) exhibited a quinolone-resistant phenotype, while significant resistance rates were also detected for gentamicin (75.6%), doxycycline (68.3%) and sulfamethoxazole-trimethoprim (63.4%). On the other hand, minocycline was the most effective antibiotic in vitro, as only 12.2% (*n* = 5) of the isolates were resistant. No statistically significant differences were detected on resistances between canine and feline samples.

The results of the MIC test were in accordance with Table 4, with a few exceptions. Initially, all isolates were susceptible to colistin and resistant to levofloxacin. All three strains which were intermediate to imipenem by the disc diffusion test (A18, A26, A28) were susceptible to both imipenem and meropenem with an MIC = 2 μg/mL by VITEK^®^2.

Moreover, four isolates resistant to amikacin by the disc diffusion method, were evaluated as intermediate (A3, A14, A40), with MIC = 32 μg/mL and susceptible (A33), with MIC = 16 μg/mL. Finally, two strains resistant to minocycline (A6,A31) were evaluated as intermediate with MIC= 8 μg/mL and one strain intermediate to gentamicin (A16) was evaluated as resistant (MIC ≥ 16 μg/mL).

Several profiles of antibiotic resistance were documented. Each specific resistance pattern and the isolates that demonstrated the respective phenotype by the disc diffusion method are listed in Table 5.

An interesting fact is that the 19 isolates, which exhibit one of the three last resistance patterns of Table 5 (No. 10, 11, and 12), could be defined as Extensively Drug Resistant (XDR), according to previously described classification [17]. Particularly, they are non-susceptible to at least one agent from all the following classes of antibiotics: aminoglycosides (gentamicin), antipseudomonal carbapenems (imipenem), antipseudomonal fluoroquinolones (ciprofloxacin), antipseudomonal penicillins + β-lactamase inhibitors (piperacillin + tazobactam), extended-spectrum cephalosporins (ceftazidime, cefepime), folate pathway inhibitors (sulphamethoxazole-trimethoprim), penicillins + β-lactamase inhibitors (ampicillin + sulbactam) and tetracyclines (doxycycline).

Additionally, the isolates are not proportionately distributed among the documented resistance patterns, since the grand majority of them are either susceptible to most drugs or XDR.

Particularly, all bacteria exhibiting an imipenem-resistant phenotype are MDR or XDR. The variation in resistance rates for each one of the antibiotics tested between carbapenem-resistant and non-resistant isolate, is presented in Table 6.

It is clearly observed that imipenem-resistant bacteria exhibit significantly higher resistance rates for all antibiotics (except quinolones, where the rate is 100% for both groups). The group size is relatively small; however, a considerable indication is provided that carbapenem resistance in the included strains is regularly co-current with resistance mechanisms against several other antibacterial agents.

### 3.3. Detection of the bla_OXA-51-like_ Gene

All isolates (41/41) were positive for the presence of the *bla*_OXA-51-like_ gene (Figure S5), in confirmation of the biochemical identification tests.

## 4. Discussion

This study indicates the presence of *Acinetobacter baumannii* as an upcoming pathogen for companion animals in Greece. To our knowledge, this is the first report of clinical cases in companion animals caused by *Abc* infections in the country.

At the same time, due to some specific properties this species possesses, such as its ability to acquire resistance against a great number of antibiotics, its tolerance in variable environments and conditions, and its capability of adherence and biofilm formation in biotic and abiotic surfaces, an important public health issue arises. Further, the XDR phenotype of a significant number of stains creates concerns about the distribution of respective bacteria through companion animals and between them and their environment.

Samples were mainly obtained from soft tissue and urinary tract infections (UTIs). This is in accordance with several recent studies of *Acinetobacter* spp. from companion animals [9,12,18,19,20,21,22]. Moreover, most isolates (29/41) were related to polymicrobial infections. Commonly co-current bacterial species included MDR Enterobacteriaceae and methicillin-resistant Staphylococci. Further, *Abc* bacteria have been regularly isolated from humans polymicrobial infections [23,24]. In a recent review, *Pseudomonas aeruginosa* was the species most commonly associated with respective cases, followed by *S. aureus*, *K. pneumoniae*, and other *Enterobacterales*. Thus, a possible beneficial interaction between these pathogens was suggested [24]. Our study provides evidence of polymicrobial *Abc* infections in dogs and cats. This could be troublesome for veterinarians concerning the virulence of the combined pathogens, the available therapeutic options and possible treatment complications.

In reference to the resistance profiles of the isolates, the rate against fluoroquinolones in this study was 100%. This is in accordance with recent relevant studies [5], indicating the wide distribution of the associated mechanisms among *Abc* complex populations.

Moreover, a high resistance rate was detected for doxycycline, while minocycline was the most effective agent tested. Doxycycline is widely used in companion animals, and therefore, the prevalence of resistant isolates under the pressure of regular administration is anticipated. Furthermore, minocycline is able to overcome several tetracycline-resistance mechanisms [5,25].

Regarding aminoglycosides, extremely high rates were documented for gentamicin, in contrast with tobramycin and amikacin, which were relatively more effective. This fact could be explained by the wide usage of gentamicin in veterinary medicine [26]. Further, these results are in accordance with previous studies [12,18].

Sulfamethoxazole-trimethoprim is a treatment option for *Acinetobacter* spp. infections, suitable for veterinary medicine. However, approximately two-thirds of the isolates in this study (26/41) were resistant. Comparable rates have been detected in several veterinary studies, especially in CR isolates [12,18,20,21].

Resistance to carbapenems is definitely of major importance. There are variable previous references of carbapenemase encoding ARGs in *Acinetobacter* spp. obtained from canine and feline specimens [12,18,20,22,27,28,29,30,31]. Most of them are associated with the *bla*_OXA-23_ gene, while other respective ARGs are only sporadically detected in pets (*bla*_OXA-58_, *bla*_NDM-1_, *bla*_IMP-1_) [9]. The prevalence of carbapenem-resistant phenotypes is significant in this study, creating concerns about the distribution of respective strains in the community. Further, Greece exhibits exceptionally high rates of carbapenem-resistant *Acinetobacter baumannii* (CRAB) over the last years, especially in hospital-acquired strains [32]. In contrast *Abc* strains producing class B carbapenemases (*bla*_NDM_) have also been detected in the country [33]. Furthermore, most of the imipenem-resistant isolates are XDR (19/20). This fact indicates that carbapenemase-encoding genes are regularly co-current with ARGs against antibiotics of different classes, such as aminoglycosides, tetracyclines, and folate pathway inhibitors. Relevant results have been provided by variable studies [9]. In *Abc* isolates of human origin, extensive research has been accomplished, revealing the ability of this bacterium to accumulate resistance determinants through the horizontal transfer of mobile genetic elements [4,34]. However, complete interpretation in our case should be carried out only by molecular investigation of these isolates’ resistome.

Dealing with infections from *Acinetobacter* spp. in pets is undoubtedly a challenge. Current research data are limited; thus therapeutic strategies are based mostly on human studies [9]. Furthermore, evaluation of the AST is based on human clinical breakpoints (Table 1) since no specific breakpoints exist for veterinary medicine [35]. Additionally, as it is clearly indicated in this study, MDR and XDR strains usually demonstrate resistance against most of the agents available for usage in animals (aminoglycosides, carbapenems, β-lactams, tetracyclines, and folate pathway inhibitors) [9]. Concerning that the only effective agents against these strains are rather critically important for human medicine (as a last resort treating options, like colistin, polymyxin B, and tigecycline) and therefore disapproved for animals [36], a Gordian knot arises for veterinarians.

Minocycline was the most effective agent, exhibiting in vitro activity, even against XDR strains. It is also a suitable alternative for methicillin-resistant Staphylococci [37], which in some cases co-existed with the *A. baumannii* isolates (polymicrobial infections). However, there are limitations in its usage regarding the site of infection and the presence of more bacterial species, especially Gram-negative.

In the future, assessing the potentialities of novel treatment approaches is inevitable. Evaluation of possible synergistic effects of currently available antibiotics or alternative, non-antibiotic treatments (bacteriophages, antimicrobial peptides, vaccines or nanoparticles) [38], could constitute available options for veterinarians.

Limited data are available about to the association of *Abc* isolates of animal origin and hospital-acquired infections. In this study, isolates were persistently obtained from samples originating from veterinary clinics for over 2.5 years (Table S1). This persistence creates concerns regarding their ability to survive and spread inside veterinary facilities. Further, as it was formerly noted, *Acinetobacter* spp. is able to survive for long periods on both biotic and abiotic surfaces, and thus, its eradication from the hospital environment is often extremely challenging [39].

About companion animals, preceding hospitalization has been associated with infections in several studies [11,19,20,21,40,41]. Distribution of highly resistant clones among veterinary clinics has also been detected [13]. Moreover, the presence of contaminated medical equipment in cases of outbreaks indicates the danger of infection during hospitalization [11,41]. Therefore, it is suggested that proper surveillance and preventive measures should be urgently established after the isolation of MDR or XDR *Abc* strains from an animal clinical sample (Figure S6) [11,42,43,44].

In reference to future research perspectives, an extensive epidemiological study would be essential to identify possible predisposing factors that enhance the involvement of highly resistant *Abc* strains in animal infections. Moreover, adopting regular and rigorous environmental sampling in veterinary facilities could provide useful information regarding the possible dissemination and persistence of «endemic» bacteria and the effectiveness of eradication measures. Finally, molecular identification and investigation of *Abc* isolates could provide sufficient data about their clonality and properties of clinical interest, such as adherence and biofilm production ability, acquired ARGs and virulence factors.

## 5. Conclusions

Bacteria of the *Abc* complex, and more specifically *A. baumannii* strains, are possible emerging pathogens for companion animals in Greece since they have been regularly isolated from infection sites in recent years. Among them, several strains exhibit MDR and XDR phenotypes, and the subsequent lack of treatment options constitutes a headache for veterinarians. The prevalence of relevant strains in pets demonstrates their wide distribution in the community and illustrates the danger of further dissemination among animals, humans, and veterinary facilities. Proper prevention and surveillance measures should be established, and further research should be accomplished to comprehend the phenomenon and restrain its advance sufficiently.

## Figures and Tables

**Table 1:** Antibacterial agents, disc content and breakpoints used in this study.

Antibacterial Agent	Disk Content (μg)	Breakpoints Used in this Study	
		Inhibition Zone (mm)	MIC (μg/mL)
Ampicillin + Sulbactam	20 + 10	S: ≥ 15, I:12–14, R: ≤ 11	---
Ceftazidime	30	S: ≥ 18, I:15–17, R: ≤ 14	---
Cefepime	30	S: ≥ 18, I:15–17, R: ≤ 14	---
Piperacillin + Tazobactam	100 + 10	S: ≥ 21, I:18–20, R: ≤ 17	S ≤ 16/4, I:32/4–64/4, R: ≥ 128/4
Imipenem	10	S: ≥ 22, I:19–21, R: ≤ 18	S ≤ 2, I:4, R: ≥ 8
Meropenem	---	---	S ≤ 2, I:4, R: ≥ 8
Amikacin	30	S: ≥ 17, I:15–16, R: ≤ 14	S ≤ 16, I:32, R: ≥ 64
Gentamicin	10	S: ≥ 15, I:13–14, R: ≤ 12	S ≤ 4, I:8, R: ≥ 16
Tobramycin	10	S: ≥ 15, I:13–14, R: ≤ 12	S ≤ 4, I:8, R: ≥ 16
Enrofloxacin	5	ES	---
Ciprofloxacin	5	S: ≥ 21, I:16–20, R: ≤ 15	S ≤ 1, I:2, R: ≥ 4
Levofloxacin	---	---	S ≤ 2, I:4, R: ≥ 8
Sulph/zole + Trimethoprim	23.75 + 1.25	S: ≥ 16, I:11–15, R: ≤ 10	S ≤ 2/38, R: ≥ 4/76
Doxycycline	30	S: ≥ 13, I:10–12, R: ≤ 9	---
Minocycline	30	S: ≥ 16, I:13–15, R: ≤ 12	S ≤ 4, I:8, R: ≥ 16
Colistin	---	---	S ≤ 2, R: ≥ 4

S: susceptible, I: intermediate, R: resistant; ES: the isolates were estimated to be resistant against enrofloxacin due to total absence of inhibition or presence of an extremely limited zone (≤12 mm) and phenotypic resistance to ciprofloxacin.

**Table 2:** Site of infection and origin of the samples included in this study.

Sample	Total Samples (%)	Canine Samples (%)	Feline Samples (%)
Soft tissue	20 (48.8%)	16 (61.5%)	4 (26.7%)
Urine	12 (29.3%)	4 (15.4%)	8 (53.3%)
Ear canal	4 (9.7%)	3 (11.5%)	1 (6.7%)
Pleural effusion	2 (4.9%)	2 (7.7%)	-
Nasal cavity	2 (4.9%)	-	2 (13.3%)
Blood	1 (2.4%)	1 (3.9%)	-
Total	41 (100.0%)	26 (100.0%)	15 (100.0%)

**Table 3:** Characteristics of the samples, the infected animals and the co-current bacteria.

Code	Sample	Origin	Gender/Age	Co-Current Isolates ^1^
A1	Soft tissue	Canine	M/4	*Ε. coli* (SDR)
A2	Pleural effusion	Canine	F/4	*Klebsiella pneumoniae* (MDR)
A3	Soft tissue	Canine	F/2	ΜRSP (MDR)
A4	Soft tissue	Feline	M/9	MRSA (MDR)
A5	Urine	Feline	M/5	ND
A6	Urine	Feline	M/9	ND
A7	Soft tissue	Canine	M/10	*Staphylococcus epidermidis*
A8	Soft tissue	Canine	F/NA	ΜRSP (MDR), *K. pneumoniae* (MDR)
A9	Soft tissue	Canine	M/12	ND
A10	Soft tissue	Canine	F/5	*Ε. coli* (SDR)
A11	Soft tissue	Canine	F/2,5	MRSA (MDR), *E. coli* (MDR)
A12	Nasal cavity	Feline	Μ/3	*Streptococcus* spp (SDR)
A13	Soft tissue	Canine	F/11	ΜRSA (MDR), *K. pneumoniae* (MDR)
A14	Urine	Feline	Μ/1	ND
A15	Soft tissue	Canine	F/2	ΜRSP (MDR)
A16	Soft tissue	Feline	Μ/1	*Enterococcus* spp (SDR), *S. epidermidis* (MDR)
A17	Pleural effusion	Canine	Μ/3	*Klebsiella oxytoca* (MDR)
A18	Soft tissue	Feline	F/1,5	*Enterobacter cloacae* (MDR)
A19	Soft tissue	Canine	F/9	*Proteus mirabilis* (MDR)
A20	Urine	Feline	Μ/NA	ND
A21	Urine	Canine	F/11	ND
A22	Urine	Feline	Μ/5	*Enterococcus* spp (MDR)
A23	Urine	Feline	Μ/8	*E. cloacae* (MDR)
A24	Nasal cavity	Feline	Μ/7	ND
A25	Ear canal	Canine	Μ/11	*Staphylococcus pseudintermedius*
A26	Urine	Canine	M/NA	*Klebsiella aerogenes* (SDR)
A27	Ear canal	Feline	F/2,5	*Bacillus* spp
A28	Ear canal	Canine	F/6	MRSP (MDR)
A29	Urine	Feline	M/3	ND
A30	Ear canal	Canine	M/12	*E. coli* (MDR)
A31	Soft tissue	Canine	F/6	*P. mirabilis* (MDR), *E. cloacae*
A32	Soft tissue	Canine	M/13	MRSP (MDR)
A33	Soft tissue	Canine	M/5	*Staphylococcus intermedius*, *P. mirabilis* (SDR)
A34	Soft tissue	Canine	M/8	*Enterococcus* spp (SDR)
A35	Urine	Canine	M/13	ND
A36	Soft tissue	Canine	M/NA	*E. coli* (MDR)
A37	Urine	Canine	F/6	ND
A38	Soft tissue	Feline	M/13	*Pseudomonas aeruginosa* (SDR)
A39	Urine	Feline	M/5	ND
A40	Soft tissue	Canine	M/3,5	*Pluralibacter gergoviae* (MDR)
A41	Blood	Canine	M/9	ND

MDR: Multi-drug resistant—an isolate that exhibits a resistant phenotype against antibiotics of three or more different classes, according to the formerly proposed criteria [17]; MRSA: methicillin-resistant *Staphylococcus aureus*; MRSP: methicillin-resistant *Staphylococcus pseudintermedius*; NA: not available; ND: not detected; SDR: single-drug resistant—an isolate that exhibits a resistant phenotype against antibiotics of one or two different classes, not counting the intrinsic resistance mechanisms of each species. ^1^ The isolates included in this section were obtained from the same sample with the selected bacteria.

**Table 4:** Resistance rates of the *Abc* bacteria included in this study by the disc diffusion method.

Antibacterial Agent	Resistant Isolates % (*n*)	Intermediate Isolates % (*n*)	Susceptible Isolates % (*n*)	Resistant Isolates in Dogs	Resistant Isolates in Cats	Fischer’s Exact Test *p*-Value
Ampicillin + sulbactam	48.8% (20)	0% (0)	51.2% (21)	14	6	*p* = 0.5204
Piperacillin + tazobactam	48.8% (20)	9.8% (4)	41.4% (17)	14	6	*p* = 0.5204
Ceftazidime	51.2% (21)	0% (0)	48.8% (20)	14	7	*p* = 0.7513
Cefepime	51.2% (21)	19.5% (8)	29.3% (12)	14	7	*p* = 0.7513
Imipenem	48.8% (20)	7.3% (3)	43.9% (18)	14	6	*p* = 0.5204
Amikacin	43.9% (18)	17.1% (7)	39% (16)	13	5	*p* = 0.3457
Gentamicin	75.6% (31)	17.1% (7)	7.3% (3)	19	12	*p* = 0.7197
Tobramycin	41.4% (17)	4.9% (2)	53.7% (22)	12	5	*p* = 0.5194
Enrofloxacin	100% (41)	0% (0)	0% (0)	26	15	*p* = 1
Ciprofloxacin	100% (41)	0% (0)	0% (0)	26	15	*p* = 1
Sulph/zole + Trimethoprim	63.4% (26)	0% (0)	36.6% (15)	17	9	*p* = 0.7485
Doxycycline	68.3% (28)	19.5% (8)	12.2% (5)	19	9	*p* = 0.4917
Minocycline	12.2% (5)	29.3% (12)	58.6% (24)	2	3	*p* = 0.3365

**Table 5:** Resistance profiles of the isolates.

No	Resistance Profile	Related Isolates
1	ENR—CIP	A2, A9, A10, A16, A19, A41
2	GEN—ENR—CIP	A12, A23, A24, A30, A38
3	GEN—ENR—CIP—SXT	A27, A36
4	ENR—CIP—SXT—DOX	A18, A26
5	GEN—ENR—CIP—DOX	A17, A25, A29
6	GEN—ENR—CIP—SXT—DOX	A28
7	AK—GEN—ENR—CIP—DOX	A33 ^1^
8	CAZ—FEP—ENR—CIP—SXT—DOX	A22
9	SAM—PIT—CAZ—FEP—IMP—ENR—CIP—SXT—DO	A21
10	SAM—PIT—CAZ—FEP—IMP—GEN—ENR—CIP—SXT—DOX	A5, A35
11	SAM—PIT—CAZ—FEP—IMP—AK—GEN—TOB—ENR—CIP—SXT—DOX	A1, A3, A7 ^2^, A8, A11, A14 ^2^, A15, A32, A34, A37, A39, A40 ^2^
12	SAM—PIT—CAZ—FEP—IMP—AK—GEN—TOB—ENR—CIP—SXT—DOX—MIN	A4, A6 ^3^, A13, A20, A31 ^3^

Antibacterial agents: AK: amikacin, CAZ: ceftazidime, CIP: ciprofloxacin, DOX: doxycycline, ENR: enrofloxacin, FEP: cefepime, GEN: gentamicin, IMP: imipenem, MIN: minocycline, PIT: piperacillin-tazobactam, SAM: ampicillin-sulbactam, SXT: sulfamethoxazole-trimethoprim, and TOB: tobramycin. ^1^ Susceptible to amikacin by MIC. ^2^ Intermediate to amikacin by MIC. ^3^ Intermediate to minocycline by MIC.

**Table 6:** Resistant rates in carbapenem-resistant and non-resistant bacteria.

Antibacterial Agent	Resistance Rate in CR Isolates Isolates % (*n*)	Resistant Rate in Carbapenem Non-Resistant Isolates % (*n*)	Fischer’s Exact *p*-Value
Ampicillin + sulbactam	100% (20/20)	0% (0/21)	*p* < 0.00001
Piperacillin + tazobactam	100% (20/20)	0% (0/21)	*p* < 0.00001
Ceftazidime	100% (20/20)	4.8% (1/21)	*p* < 0.00001
Cefepime	100% (20/20)	4.8% (1/21)	*p* < 0.00001
Amikacin	85% (17/20)	4.8% (1/21)	*p* < 0.00001
Gentamicin	95% (19/20)	57.1% (12/21)	*p* = 0.0089
Tobramycin	85% (17/20)	0% (0/21)	*p* < 0.00001
Enrofloxacin	100% (20/20)	100% (21/21)	*p* = 1
Ciprofloxacin	100% (20/20)	100% (21/21)	*p* = 1
Sulph/zole + trimethoprim	100% (20/20)	28.6% (6/21)	*p* < 0.00001
Doxycycline	100% (20/20)	38.1% (8/21)	*p* < 0.00001
Minocycline	25% (5/20)	0% (0/21)	*p* = 0.0207

CR: carbapenem resistant.

## Data Availability

The data presented in this study are available in this article.

## Supplementary Materials

The following supporting information can be downloaded at: https://www.mdpi.com/article/10.3390/vetsci10110635/s1, Figure S1: *Abc* strain growth on sheep blood agar; Figure S2. *Abc* strain growth on McConkey agar; Figure S3. Biochemical test of *Abc* strains; Figure S4. Petri dishes of the disc diffusion test; Figure S5. PCR gel electrophoresis image; Figure S6. Suggested eradication measures for veterinary facilities.
