# Supplementary material for: Antimicrobial Susceptibility Profiles of Acinetobacter baumannii Strains, Isolated from Clinical Cases of Companion Animals in Greece
Source: Vet Sci. 2023 Oct 29;10(11):635. doi: 10.3390/vetsci10110635 (PMC10674868; doi:10.3390/vetsci10110635)
Supplement: Supplementary file 1 [file vetsci-10-00635-s001.zip › Supplementary File Figures S1 to S6.pdf]

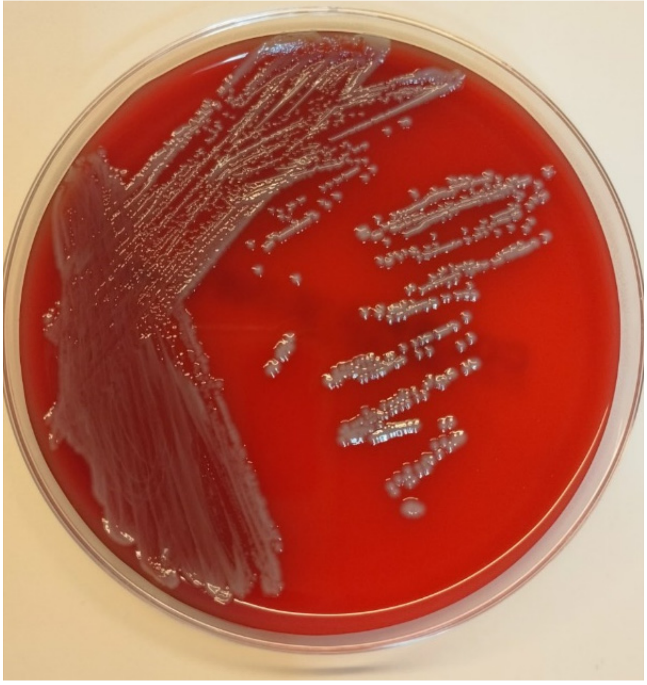

Figure S1. Growth of *Abc* complex strain (isolate number A4) on sheep blood agar.

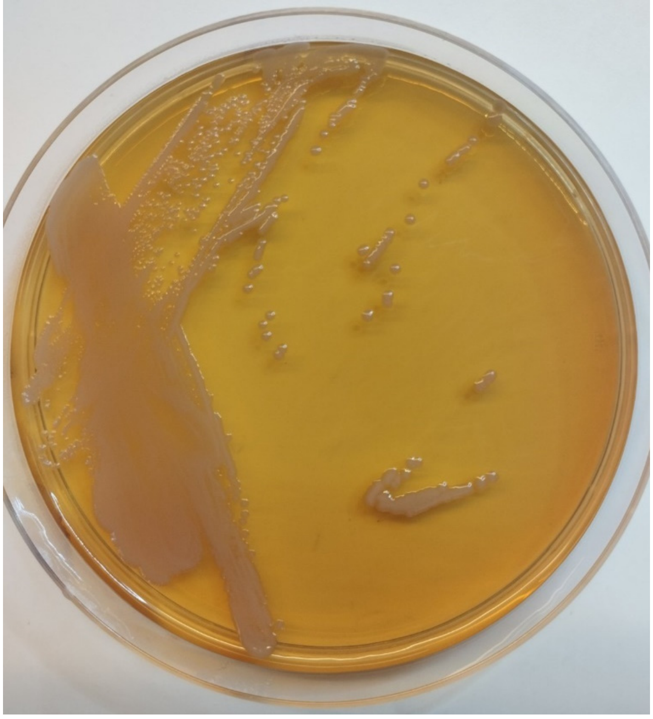

Figure S2. Growth of *Abc* complex strain (isolate number A4) on McConkey agar.

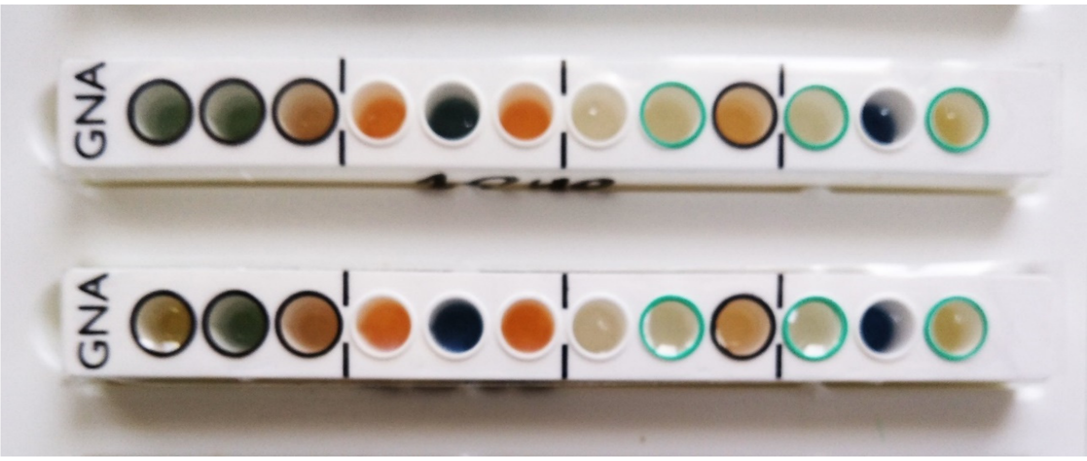

Figure S3. Biochemical tests of *Abc* complex strains (up: isolate number A6 , down: isolate number A7) using Microgen GNA-ID System, Microgen Bioproducts Ltd.

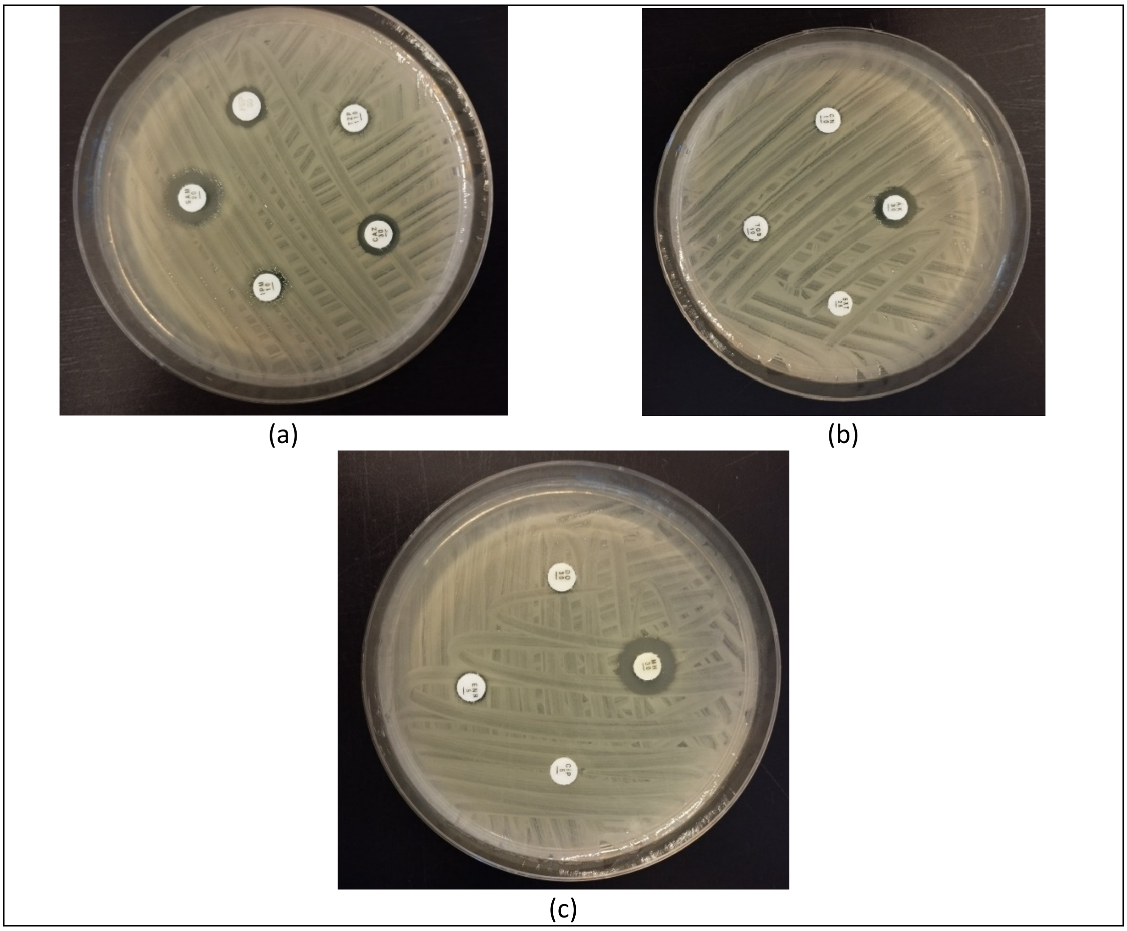

Figure S4. Petri dishes of Disc Diffusion Test. [(a) SAM: Ampicillin-Sulbactam, IPM: Imipenem, TZP: Piperacillin-tazobactam, FEP: Cefepime, CAZ: Ceftazidime; (b) CN: Gentamicin, AK: Amikacin, SXT: Sulfamethoxazole + Trimethoprim, TOB: Tobramycin; (c) DO: Doxycycline, MH: Minocycline, ENR: Enrofloxacin, CIP: Ciprofloxacin]

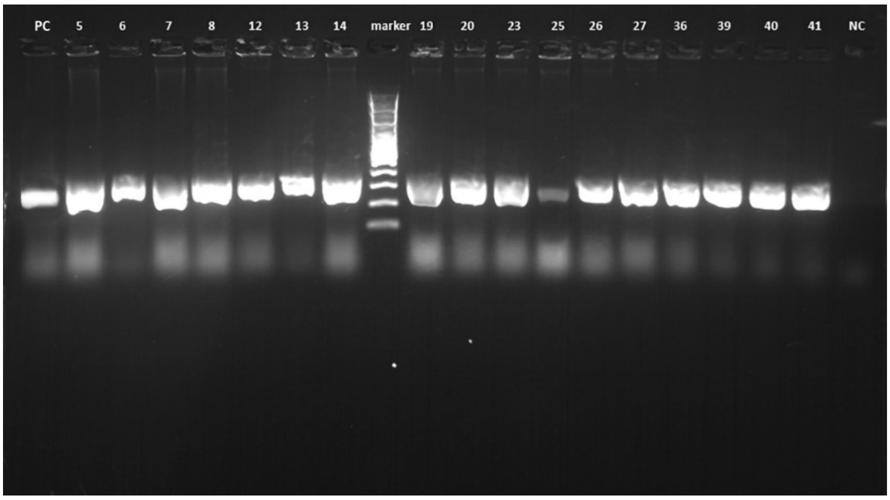

Figure S5. Indicative agarose gel electrophoresis image from a selection of samples.

**Suggested eradication measures for veterinary facilities**
• The infected animal should be isolated.<br>• Cages should be cleaned, steamed (if possible) and disinfected.<br>• Medical equipment should be dismantled and disinfected thoroughly.<br>• Disinfectants must be used at the recommended concentrations and contact times, since reduced values in these factors have been associated with bacterial survival.<br>• Environmental sampling from surfaces and equipment should be performed (usage of modified sampling techniques could be beneficial).<br>• All the aforementioned processes should be repeated if neccesary.<br>• Antibiotics in all hospitalized animals should be administrated with caution and only according to preceding culture results and susceptibility testing

Figure S6. Suggested eradication measures for veterinary facilities.
